# Pediatric Anthropometric Measurements of Dominican Republic Children

**DOI:** 10.51894/001c.164017

**Published:** 2026-07-31

**Authors:** Talon Cantrell, Imaan Hussein, Mimon Tsuji, Uday Kode, Rene Hinojosa

**Affiliations:** 1 Henry Ford Warren Hospital

## 14

### Introduction

Early childhood growth is a key indicator of health and nutrition. The World Health Organization (WHO) provides standardized growth charts for children aged 0–5 years to assess healthy development. Comparing Dominican children to WHO standards can help identify differences and guide public health policy.

### Objectives

Our goal is to identify if there is a significant difference in height and weight between children aged 0-5 years old in the Dominican Republic compared to the WHO charts. We hypothesize there is a statistically significant difference. Additionally, we will determine whether a gender difference exists.

### Methods

Data was collected from a total of 40 children, ranging from 3 months to 5 years old, from 3 different Bateys (underserved rural communities) in the Dominican Republic. Measurements of weight, height, head circumference, mid upper arm circumference, tricep and subscapular skinfold measurements were obtained. Z-values for height and weight were plotted against age to identify their distribution considering the WHO standards. Then, Bland-Altman plots assessed the degree of agreement or bias between measured and expected values provided by WHO standards. A t-test was performed to test the significance of gender differences.

### Results

Based on WHO standards, 11.5% of children in the Bateys would be classified as moderate or severely underweight, and 7.7% would be considered overweight. For height, 10.3% of children were considered of tall stature while 3.4% are of short stature. 86.3% are within normal range by WHO standards. Differences between genders for height and weight were not statistically significant with t-values of -0.512 and 0.779 for weight and height respectively (*p* > 0.05 for samples of unequal variance). The Bland-Altman plot of height displays a bias of 0.73cm over the expected height while the Bland-Altman plot of weight differences shows a bias of 0.36kg over the expected weight.

### Conclusion

The presence of bias in the Bland-Altman suggests that the WHO charts may not accurately capture the growth of children in the Bateys. A significant gender difference was not observed. However, this may result from the small sample size (n=40). Further analysis with increased sample size should be conducted.

